# HSP90β as a potential prognostic biomarker in urothelial carcinoma: insights from NF-κB, IL-8, and heat-shock protein expression

**DOI:** 10.1007/s12094-025-04181-9

**Published:** 2025-12-23

**Authors:** Jędrzej Borowczak, Edyta Podemska, Małgorzata Drobnik, Aleksandra Gawrych, Łukasz Szylberg, Magdalena Bodnar

**Affiliations:** 1 Faculty of Medicine, Bydgoszcz University of Science and Technology, Aleje Prof. S. Kaliskiego 7, 85-796 Bydgoszcz, Poland; 2 Clinical Department of Oncology, Oncology Centre – Prof. Franciszek Łukaszczyk Memorial Hospital, 85-796 Bydgoszcz, Poland; 3 Department of Obstetrics, Gynaecology and Oncology, Collegium Medicum Nicolaus Copernicus University, 85-168 Bydgoszcz, Poland; 4 Department of Tumor Pathology and Pathomorphology, Oncology Centre – Prof. Franciszek Łukaszczyk Memorial Hospital, 85-796 Bydgoszcz, Poland; 5 Chair of Pathology, Dr Jan Biziel Memorial University Hospital, 85-168 Bydgoszcz, Poland

**Keywords:** Urothelial carcinoma, Heat, Shock proteins, HSP90, IL-8, NF-κB, Biomarker

## Abstract

**Background:**

Urothelial carcinoma (UC) is a common malignancy with high recurrence rates and limited prognostic markers. Inflammation and stress-response pathways are implicated in tumor progression, with NF-κB, interleukin-8 (IL-8), and heat shock proteins (HSP90α/β) playing key roles in survival signaling and treatment resistance. We evaluated the expression of these proteins in UC and assessed their associations with clinicopathological features.

**Methods:**

Formalin-fixed, paraffin-embedded samples from 102 UC patients (168 tumor sections) and 23 controls were analyzed by immunohistochemistry for NF-κB, IL-8, HSP90α, and HSP90β. Expression was semi-quantitatively scored using the immunoreactive score (IRS) and correlated with clinicopathological features using non-parametric tests.

**Results:**

Nuclear NF-κB expression was significantly higher in UC compared with normal urothelium and correlated with tumor stage and nodal involvement. IL-8 was expressed in 53.6% of tumors and 48.8% of adjacent infiltrates but showed no association with grade, stage, or lymph node status; a weak positive correlation with inflammatory infiltrate was observed. Both HSP90 isoforms were upregulated in UC relative to controls, but only HSP90β correlated with grade, stage, and lymph node status. No significant correlations were found between NF-κB, IL-8, and HSP90 expression.

**Conclusions:**

HSP90β expression shows potential as a prognostic biomarker and therapeutic target in UC. Although NF-κB and IL-8 did not demonstrate consistent prognostic value, their patterns highlight the role of inflammatory signaling in tumor biology. These findings warrant validation in larger, more diverse cohorts and support exploration of HSP90-targeted strategies in UC.

**Supplementary Information:**

The online version contains supplementary material available at 10.1007/s12094-025-04181-9.

## Introduction

Urothelial carcinoma (UC) is the sixth most common cancer in the United States, with an estimated 81,180 new cases and 17,100 deaths annually [1]. The disease primarily affects men in their seventh decade of life. Tobacco smoking is the primary risk factor, contributing to the development of the disease in up to 50% of cases [2, 3]. UC encompasses two biologically and molecularly distinct types: low-grade papillary carcinoma and high-grade carcinoma, which can present as carcinoma in situ or invasive disease [4]. Over 70% of patients are diagnosed at a non-invasive stage (stages Ta, Tis, T1), while the remaining 30% are diagnosed at an invasive stage (pT2-T4) or with metastatic disease. Unfortunately, despite appropriate treatment, many patients experience recurrence, leading to unresectable, locally advanced, or metastatic tumors [5].

Genomic instability—characterized by an increased frequency of point mutations, deletions, amplifications, chromosomal rearrangements, and loss of heterozygosity—is considered one of the major drivers of UC progression [6]. To date, two main patterns of UC-associated genetic instability have been identified: *HRAS* and *FGFR3* mutations, typically found in low-grade papillary tumors, and alteration of tumor suppressor pathways (e.g. *RB1* and *TP53*) in high-grade invasive carcinomas [4, 7]. Under stressful conditions, cancer cells may acquire new mutations and copy-number alterations, promoting the selection of cells less susceptible to drug-induced cytotoxicity. This phenomenon is contributes to relapse, drug resistance, and poor patient outcomes. Intratumoral heterogeneity further facilitates the persistence of checkpoint inhibitor-resistant subclones, accelerating disease progression [8, 9].

Several theories attempt to explain the process of tumorigenesis in UC. One proposes that genetic mutations induce genetic instability, leading to oncogene activation and loss of tumor suppressor genes [4]. This cascade promotes the release of inflammatory mediators and the formation of an inflammatory microenvironment. Conversely, chronic inflammation itself may act as an independent carcinogenic factor, as tumors modulate the immune system through cytokines, chemokines, and growth factors [10, 11]. Among these, dysregulated CXC chemokines, particularly interleukin-8 (IL-8), are pivotal [12]. IL-8, secreted by activated monocytes and macrophages, activates neutrophils, enhances angiogenesis, and signals through the transcription factor NF-κB [12, 13]. NF-κB proteins (p50, p52, p65/RelA, c-Rel, RelB) remain cytoplasmic in resting cells but, upon inflammatory stimulation, translocate to the nucleus to induce gene expression [11]. Through this pathway, NF-κB upregulates DNA repair proteins (e.g., BRCA2, MnSOD), chaperones (Hsp90α), and anti-apoptotic factors (Bcl-2), thereby promoting tumor cell survival and drug resistance [14, 15].

Heat shock protein 90 (Hsp90) is a family of molecular chaperones with essential cytoprotective functions. It exists in two isoforms: the major stress-inducible Hsp90α, which readily forms dimers, and the constitutively expressed Hsp90β, which dimerizes less efficiently. Both isoforms are crucial for maintaining cellular homeostasis, DNA repair, transcriptional regulation, and chromatin remodeling [16, 17]. In response to cellular stressors, such as heat, chemical damage, or anticancer therapies, Hsp90 expression increases, leading to Akt activation and inactivation of pro-apoptotic proteins, such as Bcl-2, Bad, and caspase 9 [18, 19]. Akt also inhibits IκB kinase, further sustaining NFκB signaling [20, 21]. These properties establish Hsp90 as a central regulator of stress adaptation and drug resistance in cancer.

The aim of our study was to evaluate the expression of IL-8, NF-κB, HSP90α, and HSP90β in urothelial carcinoma, to investigate correlations between these pathways, and to assess whether a proinflammatory tumor microenvironment influences HSP activity and cancer response to cellular stress.

## Materials and methods

### Sample acquisition

This study included 168 urothelial carcinoma samples collected from 102 patients with diagnosed urothelial carcinoma and treated in the Department of Urology between 2009 and 2015. The research group was comprised of 23 women and 79 men, with a median patients age of 65 years (ranging from 33 to 92 years). The control group included 23 normal urothelial tissue samples present in the resection specimens. Clinical data, including age, sex, and disease advancement, were obtained (Table 1). The study followed the Declaration of Helsinki, and the protocol was approved by the local Bioethics Committee (KB 470/2012).

**Table 1:** Basic patient characteristics

Clinical data		*n* (%)
Patients		102
Samples		168
Median age		65 years (range 33–82)
Age	≤ 65	48 (47.1%)
	> 65	54 (52.9%)
Sex	Female	23
	Male	79
Grade	G1	7 (7%)
	G2	57 (56%)
	G3	32 (31%)
Stage	pTa	19 (19%)
	T1	15 (15%)
	T2	23 (23%)
	T3	25 (25%)
	T4	20 (20%)
Lymphnodesinvasion	N0	60 (59%)
	N1	8 (7.1%)
	N2	13 (11.6%)
	N3	4 (3/6%)
	NX	17 (16%)
Metastasis	M0	102 (100%)
	M1	0 (0%)

### Tissue microarrays

Histopathological specimens stained with H&E were initially screened to determine the representative tumor and normal tissue areas. The archival formalin-fixed, paraffin-embedded (FFPE) samples were mixed with wax to create soft blocks of uniform height. Tissue microarrays were obtained by cutting representative tissue fragments from the “donor” block using the TMA Master 1.14 SP1 device from 3D HISTECH. Then, 3 μm thick specimens were cut from TMA blocks using a manual rotary microtome (Accu—Cut® SRM™ 200, Sakura). The paraffin sections were heated at 50–60 °C and HE staining was performed to verify the correctness of the collected tissue material before immunohistochemistry.

### Immunohistochemistry

**Fig. 1:**
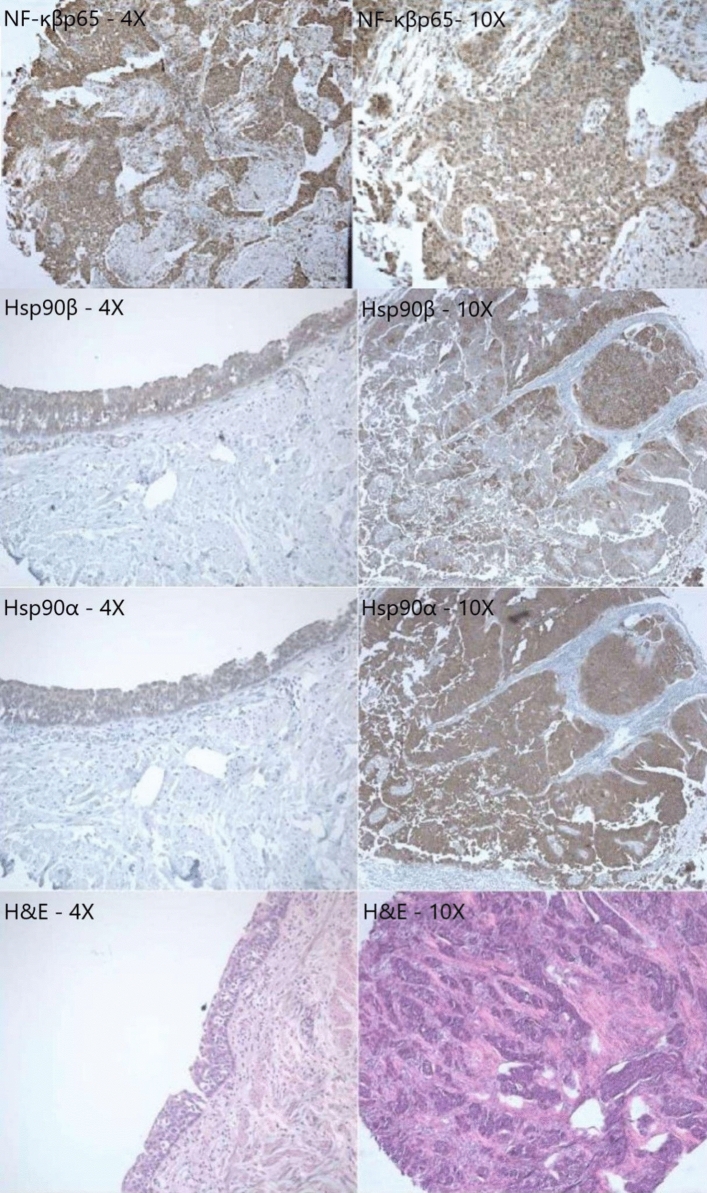
Representative cross-section staining of Hsp90α, Hsp90β, NF-κβp65, and H&E

A retrospective immunohistochemical analysis of Hsp90α, Hsp90β, NF-κβp65, and IL-8 comprised 168 FFPE tissue blocks (Fig. 1). In brief, 3 μm thick sections of the tissue arrays were baked for 1 h at 60 °C before xylene deparaffinization and subsequent rehydration through graded ethanol (99.8, 96, 90, and 80%). Tissue sections were incubated with a primary rabbit monoclonal anti-Hsp90α (1:100, 30 min, Ab79849, Abcam), anti-Hsp90β (1:2000, 30 min; Ab53497, Abcam), anti-NF-κβp65 (1:500, 16 h, Ab31481, Abcam), and anti-IL-8 antibodies (1:100, 16 h, Ab18672, Abcam) (Fig. 1). The visualization of primary antibodies was performed using the UltraView Universal DAB Detection Kit (Roche Diagnostics/Ventana) followed by color development using 3,3-diaminobenzidine. The slides were counterstained with Hematoxylin II for 12 min and Bluing Reagent for 4 min; then the tissue sections were dehydrated in increasing ethanol concentrations (80, 90, 96, and 99.8%), cleared in xylenes (I–IV), and mounted using a mounting medium.

### Image acquisition and expression analysis

Clinical data were blinded and images of each sample were captured using a Nikon Eclipse E400 optical microscope at × 4, × 10, and × 20 magnification with a color video camera attached to a computer system. The presence of antigen-specific antibody-secondary antibody complexes was indicated by the brown color. The presence of inflammatory infiltrate was assessed on H&E-stained slides using a nominal scale (0—no inflammatory infiltrate; 1—inflammatory infiltrate present). The expressions of NF-κβp65, Hsp90α, and Hsp90β were analyzed in cancer tissues using the semi-quantitative, modified Remmele-Stegner scale (0–12), calculated as the product of the intensity of expression and the percentage of stained cells (Table 2). IL-8 expression was assessed in urothelial cancer tissue and inflammatory infiltrate using nominal (0—no IL-8 expression; 1—IL-8 expression present). The result was considered positive if there were > 10 positive cells in a single field of vision at × 20 magnification.

**Table 2:** Immunohistochemical reaction scale by Remmele-Stegner

		% of cells with positive expression [PP]	Expressionintensity [SI]
IRS scale [PP] x [SI]	0	No cells	No expression
	1	< 10% of cells	Weakexpression
	2	10–50% of cells	Moderateexpression
	3	50–80% of cells	Strongexpression
	4	80–100% of cells	Verystrongexpression

### In silico analysis

TCGA GDC BLCA cohort clinical data were accessed through the cBioPortal [22]. USCS XENA was used to acquire normalized FPKM expression of HSP90AB1, RELA, and CXCL8 from the GDC TCGA Bladder Cancer (BLCA) dataset [23]. The FPKM cut-offs were set at 8.377 for HSP90AB1, 4.493 for RELA, and 4.427 FPKM for CXCL8.

### Statistical analysis

All statistical analyses were performed using Statistica version 13.3 (Statsoft) and Microsoft Excel 2019. Variables were tested for normality by the Shapiro–Wilk test. Gene expression data were analyzed using nonparametric tests. The intergroup comparisons used the Mann–Whitney U test or the ANOVA Kruskal–Wallis test. The correlations between protein expressions and clinicopathological features of urothelial carcinoma were evaluated using Spearman's rank correlation coefficient. The assumption of the Cox proportional hazard models was tested using the proportional hazard test and Schoenfeld’s rests. The p-value was considered statistically significant if < 0.05.

## Results

### NF-κβp65 is overexpressed in urothelial carcinoma

**Fig. 2:**
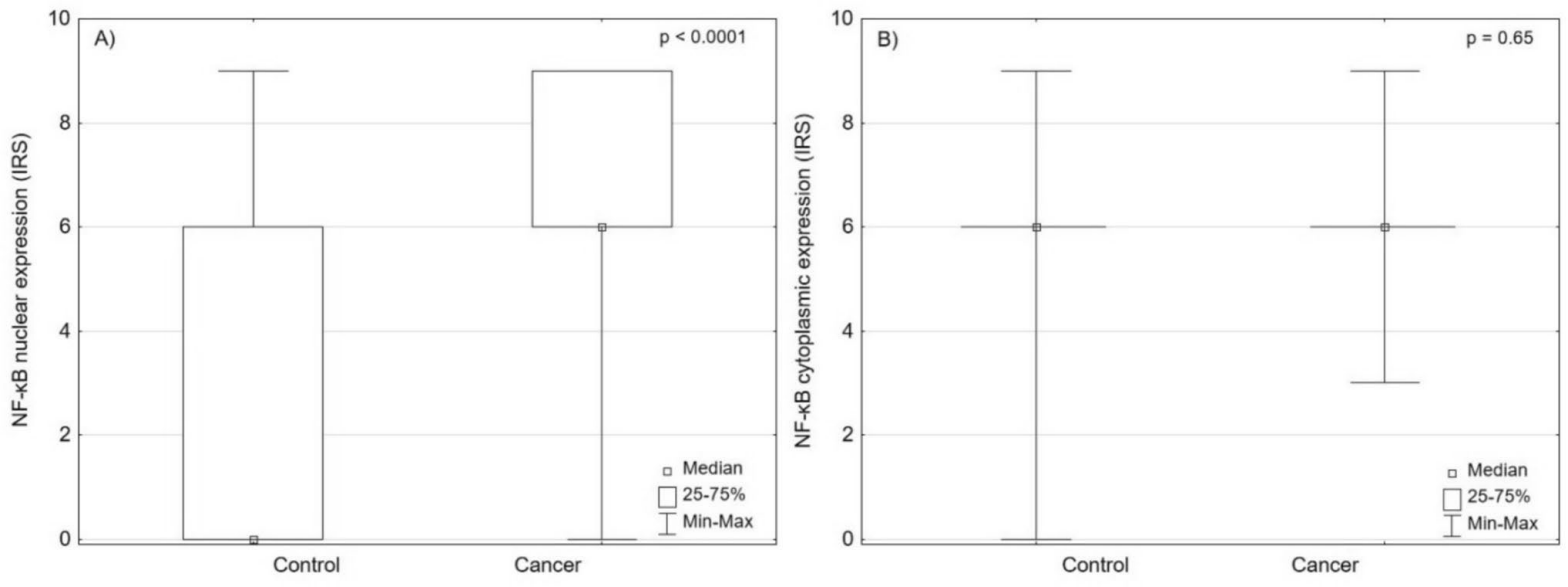
The differences in NF-κB expression between cancer and normal urothelial tissues, **A** nuclear staining (*p* = 0.0003), **B** cytoplasmic staining (*p* = 0.65)

**Fig. 3:**
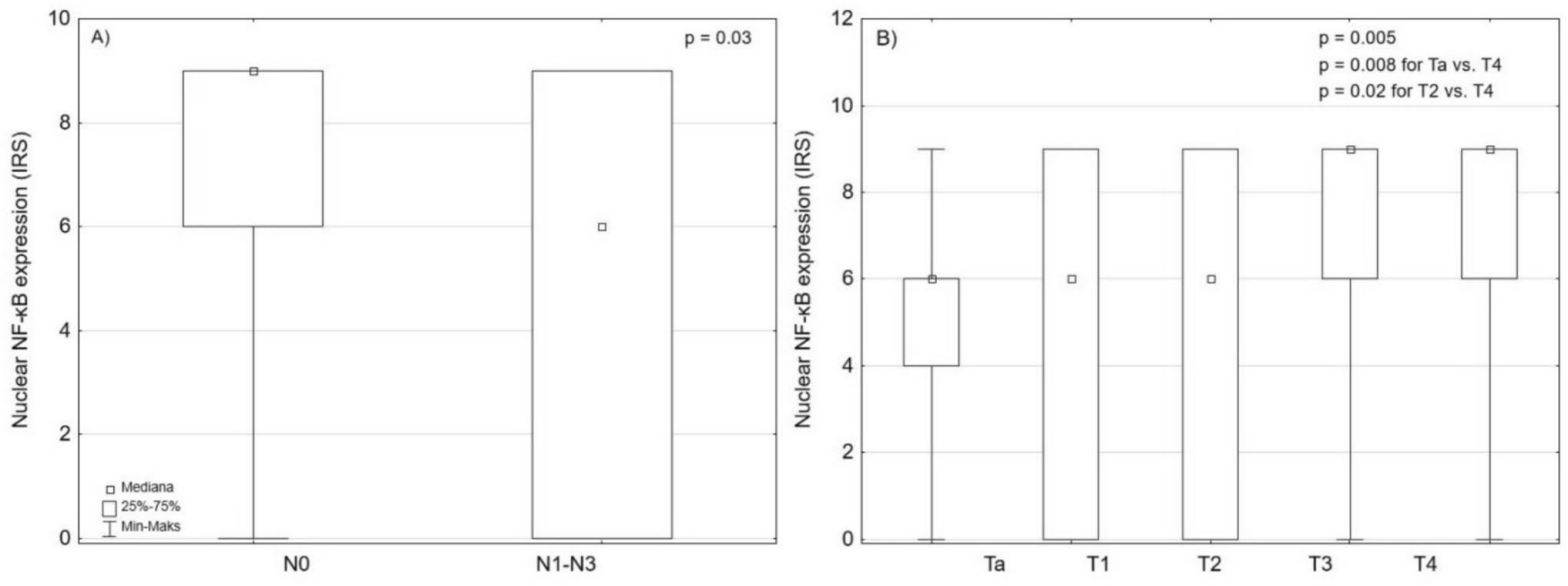
The differences in NF-κB expression depending on (**A**) lymph node status (*p* = 0.02), and **(B)** tumor stage (*p* = 0.0005)

First, we analyzed the expression of NF-κβp65 in the urothelial carcinoma tissues. The median nuclear NF-κB expression was significantly higher in urothelial carcinoma compared to normal urothelial tissue (IRS 6 vs. 0, respectively; *p* = 0.0003). There were no differences between median cytoplasmic NF-κB expression between cancer and normal tissue (*p* > 0.05). Therefore, further analysis was conducted using only nuclear expression of NF-κB (Fig. 2). Nuclear expression of NF-κB increased with tumor stage and was higher in cases with lymph node involvement (Table 3 and Fig. 3).

**Table 3:** Nuclear NF-κB expression depending on clinicopathological features of urothelial carcinoma

Variable	Total (*n*)	Nuclear NF-κB expression (IRS)	Q1	Q3	*p*-value
Ta	21	6 (range 0–9)	6	9	***p* = 0.003**
T1	20	6 (range 0–9)	0	9	
T2	38	6 (range 0–9)	0	9	
T3	48	9 (range 0–9)	4.5	9	
T4	41	9 (range 0–9)	6	9	
N0	94	6 (range 0–9)	4	9	***p* = 0.02**
N1-N3	48	9 (range 0–9)	4	9	
G1	9	6 (range 0–9)	6	9	***p* = 0.72**
G2	89	6 (range 0–9)	6	9	
G3	57	6 (range 0–9)	0	9	

*T*—stage; *N*—lymph node status; *G*—histological grade; *Q*—quartile; Statiscically significant results are in bold.

### Inflammatory infiltrate and Il-8 expression in urothelial carcinoma

In the next step, we assessed the presence of inflammatory infiltrate and IL-8 expression. The inflammatory infiltrate was present in 136/168 (81%) samples. IL-8 was expressed in 90/168 (53.6%) urothelial cancer tissues and in 82/168 (48.8%) adjacent inflammatory infiltrates. We found a weak positive between the positive expression of IL-8 in urothelial cancer and inflammatory infiltrate, and the presence of inflammatory infiltrate (*r* = 0.35, *p* < 0.05). The prevalence of IL-8 positive expression in cancer and inflammatory infiltrate did not correlate with tumor grade, stage, nor lymph node status (Table 4). No significant differences in NF-κB levels were observed according to IL-8 status, either in tumor cells or inflammatory infiltrates (*p* > 0.05).

**Table 4:** Prevalence of inflammatory infiltrate urothelial carcinoma and IL-8 positive expression in bladder cancer and inflammatory infiltrate

Variable	Inflammatory infiltrate	IL-8 expression in cancer	IL-8 expression in inflammatory infiltrate
Ta	14/21	9/21	12/14
T1	17/20	12/20	12/17
T2	36/38	14/48	13/36
T3	40/48	28/48	26/40
T4	29/41	15/41	15/29
N0	79/95	44/94	45/79
N1-N3	37/48	20/48	20/37
G1	6/9	3/9	5/6
G2	69/89	41/89	41/69
G3	49/57	30/57	28/49

*T*—stage; *N*—lymph node status; *G*—histological grade; *Q*—quartile

### The analysis of Hsp90α and Hsp90β expression patterns in urothelial carcinoma

**Fig. 4:**
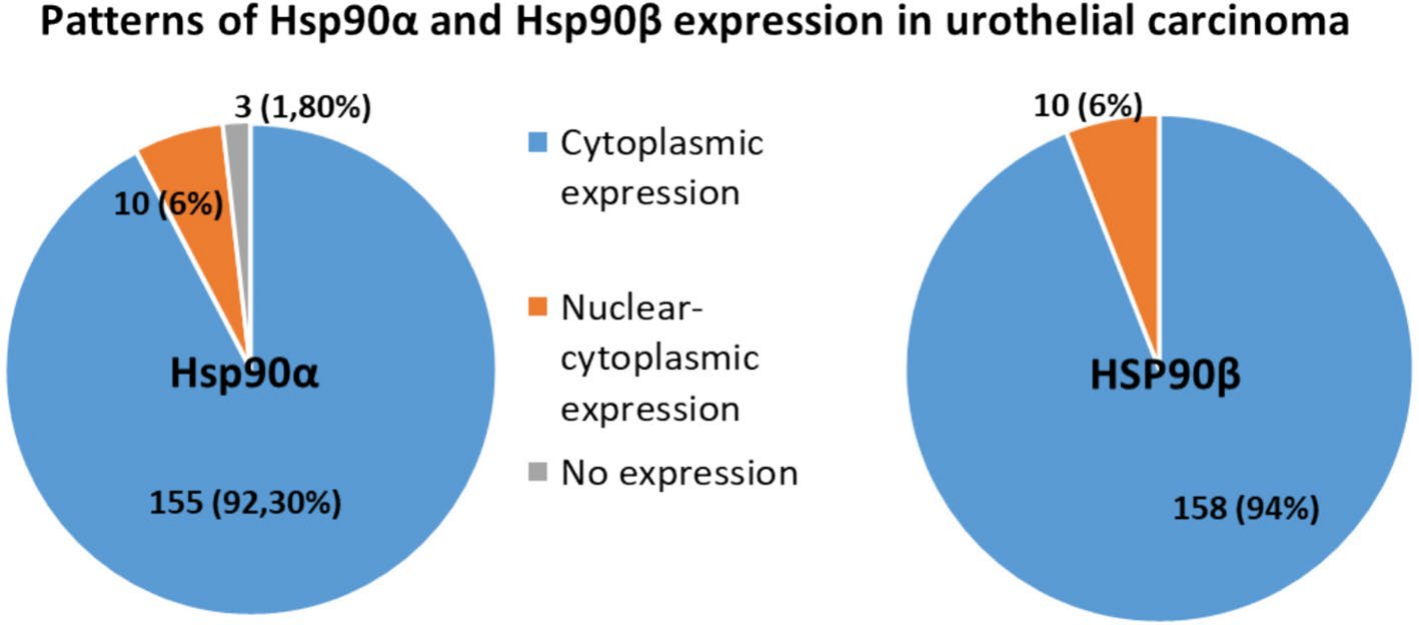
Patterns ofHsp90α and Hsp90β expression in urothelial carcinoma

**Fig. 5:**
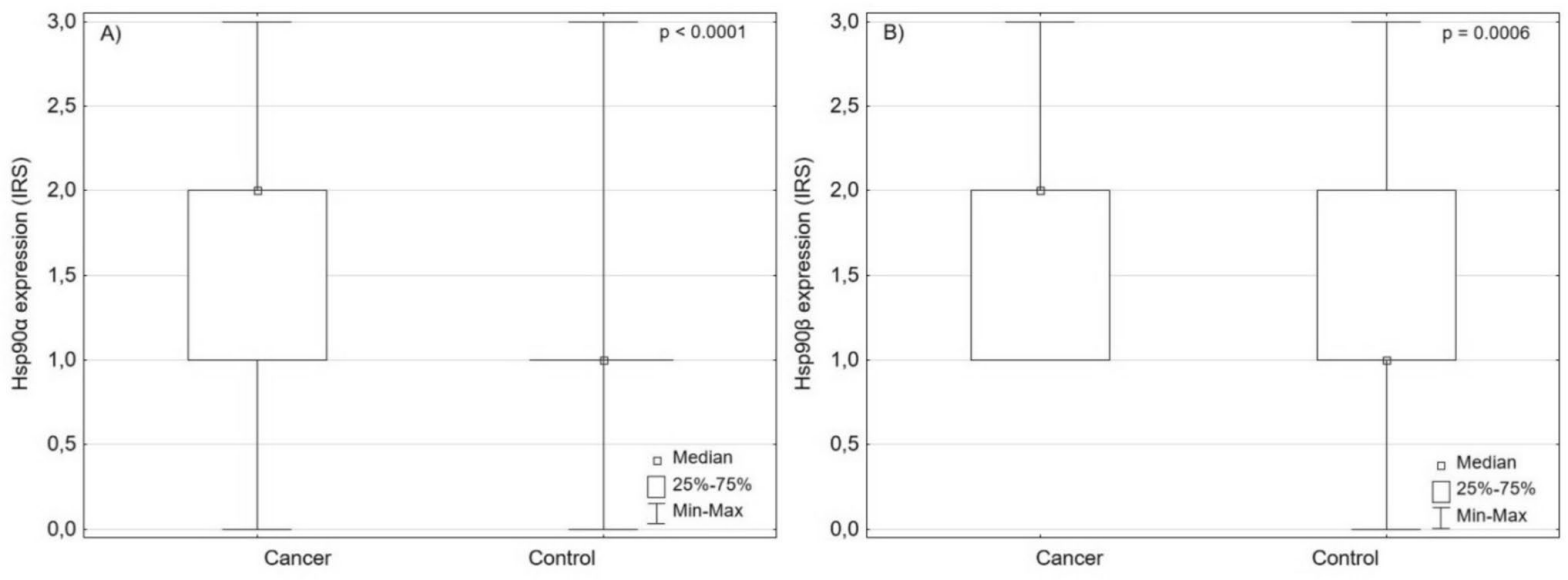
**A** Hsp90α and **B** Hsp90β are overexpressed in urothelial carcinoma compared to urothelial epithelium

Of 168 examined specimens, positive Hsp90α expression was detected in 165 (98.2%) samples and positive Hsp90β expression in 168 (100%) samples. Both groups showed predominantly cytoplasmic staining pattern, which were found in 92.3% of samples stained with Hsp90βα and 94% of samples stained with Hsp90β. Mixed nuclear-cytoplasmic staining pattern was found in 6% (10 cases) of each sample (Fig. 4). Hsp90α and Hsp90β were overexpressed in urothelial carcinoma compared to urothelial epithelium (*p* < 0.05, Fig. 5).

**Fig. 6:**
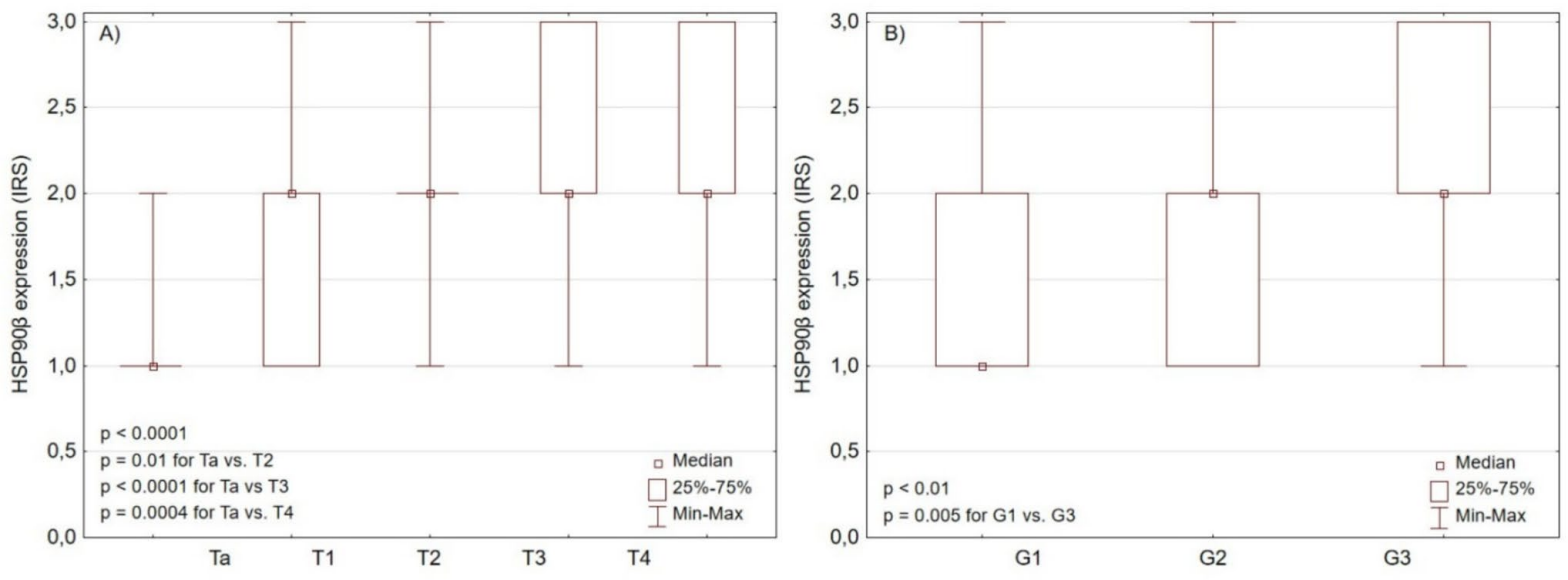
Hsp90β expression depending on **A** tumor stage and **B** tumor grade

Hsp90β levels were positively correlated with tumor stage, grade, and lymph node involvement (Fig. 6). We found no association between Hsp90α expression and clinicopathological features of urothelial carcinoma (Table 5). Furthermore, we found no correlation between the expressions of Hsp90α, Hsp90β, NF-κB, and IL-8. HSPs levels were also independent of the presence of inflammatory infiltrate within the tumor.

**Table 5:** Hsp90α and Hsp90β expression depending on clinicopathological features of urothelial carcinoma

Variable	Total (*n*)	Hsp90α	Q1	Q3	*p*-value	Hsp90β	Q1	Q3	*p*-value
Ta	21	2 (range 1–2)	1	2		1 (range 1–2)	1	1	
T1	20	2 (range 1–3)	1	2		2 (range 1–3)	1	2	
T2	37	2 (range 1–3)	1	3	*p* = 0.26	2 (range 1–3)	2	3	***p* = 0.001**
T3	48	2 (range 0–3)	1	2		2 (range 1–3)	2	3	
T4	41	2 (range 0–3)	2	3		2 (range 1–3)	2	3	
N0	93	2 (range 0–3)	1	2	*p* = 0.07	2 (range 1–3)	1	2	***p* = 0.005**
N1-N3	48	2 (range 0–3)	1	2		2 (range 1–3)	2	3	
G1	9	2 (range 1–3)	1	2	*p* = 0.16	1 (range 1–3)	1	2	***p* = 0.004**
G2	89	2 (range 0–3)	1	2		2 (range 1–3)	1	2	
G3	56	2 (range 0–3)	1	2		1 (range 1–3)	2	3	

Statistically significant results are in bold

### HSP90AB1 expression analysis in the TCGA GDC BLCA cohort

We assessed the expression HSP90AB1, RELA, and CXCL8 in the 403 bladder cancer samples from the TCGA GDC BLCA cohort using the XENA browser (Supplementary Table 1). We also extracted the subset of matched normal urothelial samples from the TCGA-BLCA cohort to enable direct tumor–normal expression comparisons. Tumor samples exhibited significantly higher levels of HSP90AB1 than normal urothelial tissues (9.13 vs. 8,56 FPKM, *p* = 0.008; Supplementary Fig. 1). We found no differences in RELA and CXCL8 levels between tumors and normal urothelial tissues.

We found no correlation between the expressions of HSP90AB1 and clinicopathological features of bladder cancer, such as tumor stage, lymph node involvement, patients' sex, race, and clinical stage (all *p* > 0.05, Supplementary Table 2). In contrast, CXCL8 expression was nominally higher in female patients compared with male patients (*p* = 0.048), and RELA expression also demonstrated higher levels in females (*p* = 0.036); however, these associations did not remain significant after adjustment for multiple testing.

Next, we used the Ensembl gene ID from TCGA to map the TCGA RNA-seq data. The samples were dichotomized into low and high HSP90AB1 expression groups with an FPKM (number of fragments per kilobase of exon per million reads) cutoff value set at 8.377. BLCA patients with high HSP90AB1 expression had significantly shorter overall survival (*p* = 0.03) than patients with low HSP90AB1 expression (Supplementary Fig. 2).

In the univariate Cox proportional hazards analysis, older age, lymph node involvement, and high HSP90AB1 expression were significantly associated with shorter overall survival. In the multivariate model, all three variables remained independent prognostic factors (Table 6). The proportional hazards assumption was verified using Schoenfeld residuals and was met for all covariates as well as for the model overall (all *p* > 0.05).

**Table 6:** Univariate and multivariate analysis of the TCGA GDC BLCA cohort (*n* = 403)

Variable	Univariate analysis			Multivariate analysis		
	HR	95% CI	*p*-value	HR	95% CI	*p*-value
Age (per year)	1.04	[1.02–1.05]	** < 0.001**	1.04	[1.02–1.06]	** < 0.001**
Sex (Male vs. **Female**)	1.13	[0.81–1.56]	0.48	–	–	–
Clinical stage (I-II vs. **III-IV**)	0.7	[0.27–1.82]	0.47	–	–	–
Nodes (N0 vs. **N1-N3**)	2.21	[1.61–3.03]	** < 0.001**	1.83	[1.01–3.3]	** < 0.001**
HSP90AB1 (Low vs. **High**)	1.9	[1.06–3.42]	**0.03**	2.13	[1.55–2.92]	**0.044**
CXCL8 (Low vs. **High**)	0.98	[0.73–1.31]	0.88	–	–	–
RELA (Low vs. **High**)	0.87	[0.64–1.17]	0.34	–	–	–

## Discussion

In our study, nuclear NF-κB expression was significantly higher in urothelial carcinoma compared to normal urothelium and positively correlated with tumor stage and lymph node involvement (Figs. 3, 4). These findings highlight the potential role of NF-κB in disease progression. Other associations have been reported by other groups: overexpression of RelA has been linked to higher grade, advanced stage, and poor survival bladder carcinoma, while nuclear p65 localization has been identified as an independent prognostic factor in patients treated with bladder-preserving therapy [24–26]. However, not all studies confirms this relationship; Degoricija et al. found no correlation between NF-κB activity and tumor infiltration depth [27].

In addition, in silico analyses have suggested that NF-κB subunit distribution may vary with tumor differentiation: poorly differentiated carcinoma show nuclear accumulation of both p65 and p50, whereas well-differentiated tumors display nuclear p65 only, with RelA largely retained in the cytoplasm [28]. These findings imply the involvement of alternative NF-κB activation mechanisms depending on tumor phenotype, with potential therapeutic implications. In patients with muscle-invasive bladder cancer, nuclear RelA overexpression has been linked to resistance to neoadjuvant chemotherapy and chemoradiotherapy [24]. NF-κB is also critically involved in regulating inflammatory responses within the tumor microenvironment, which may drive both genetic and epigenetic alterations [11, 29].

One of its key transcriptional targets is IL-8, which in UC is associated with increased angiogenesis, immune cell infiltration, and tumor progression [30]. IL-8 can also reinforce NF-κB signaling through AKT activation, creating a positive feedback loop that promotes tumor growth, as reported in prostate cancer models [31]. In our study, IL-8 overexpression was confirmed in tumor specimens. However, NF-κB levels did not differ between IL-8–positive and –negative cases, either in tumors parenchyma or inflammatory infiltrates. IL-8 expression was not associated with tumor stage or grade, although a weak positive correlation was observed with the presence of inflammatory infiltrate, possibly reflecting IL-8–driven recruitment of immune cells. These findings may be influenced by biological heterogeneity, limiting the interpretability of IHC results.

In contrast to previous reports, IL-8 overexpression in our study was not associated with poor clinical prognosis or disease recurrence [32]. The divergence between our results and previous reports linking IL-8 to poor clinical outcomes and treatment resistance may stem from cohort differences (e.g., disease stage, follow-up) and the inherent limitations of IHC, which does not capture protein functionality [12, 32–34]. Furthermore, the dual nature of IL-8’s effects may vary with the immunological microenvironment of individual tumors. Further research using functional and quantitative approaches is warranted to clarify its prognostic and therapeutic potential.

Take together, those results suggest that IL-8 plays a dual role in UC pathogenesis. On one hand, it acts as a proinflammatory and proangiogenic factor, activating MAPK, JAK2, and FAK pathways, and promoting leukocyte recruitment [35]. On the other hand, IL-8 can stimulate antitumor response by attracting and activating immune cells, and under specific conditions may facilitate tumor suppression through immune-mediated elimination of cancer cells.

Given the established role of heat shock proteins (Hsp90) in NF-κB activation and inflammatory signaling, we next analyzed Hsp90 expression in UC. Both of Hsp90α and Hsp90β levels were significantly elevated in UC compared to normal urothelium. Notably, only Hsp90β expression showed a significant association with tumor grade, clinical stage, and lymph node involvement. Both isoforms displayed predominantly cytoplasmic staining, with mixed nuclear localization in a minority of cases. Although not analyzed separately in our study, subcellular localization may influence Hsp90 activity and merits further investigation.

Although Hsp90 overexpression in UC is well documented, most studies focus on total Hsp90 or only one single isoform [17]. Emerging data suggest distinct biological roles for Hsp90α and Hsp90β [16]. Hsp90α, a stress-inducible isoform, supports cell motility and stress adaptation, whereas Hsp90β is constitutively expressed and stabilizes key oncogenic proteins, such as p53 or FAK [36, 37]. This, Hsp90βexpression may reflect stable, tumor-promoting molecular alterations rather than transient microenvironmental responses, which could explain its stronger correlation with adverse clinicopathological features.

In the final phase of the study, we analyzed co-expression of NF-κB, IL-8, and HSPs to explore their potential interplay. NF-κB activation promotes proinflammatory cytokine expression, including IL-6 and IL-8, and is modulated by STAT3 and AKT pathways [21, 38]. IL-8, in turn, reinforces NF-κB signaling through AKT activation, creating a feed-forward loop that supports tumor growth [31]. Despite the well-established interactions, we found no correlation between NF-κB, IL-8, and Hsp90 expressions in our study cohort, suggesting biological heterogeneity.

Nevertheless, Hsp90 is known to enhance signaling and inflammatory cytokine production, and its overexpression has been linked to chemoresistance in other malignancies. For example, in ovarian carcinoma, Hsp90-overexpressing cells exhibited resistance to cisplatin and paclitaxel via AKT/GSK3β/β-catenin activation [19]. These observations point to potential crosstalk between Hsp90, NF-κB, and IL-8 that may also be relevant in UC. Although some supporting evidence comes from other tumor types, the conserved involvement of key pathways suggests that similar processes may occur in bladder cancer. A deeper understanding of the mechanism will require further mechanistic studies.

Our study has several limitations that should be acknowledged. The cohort was relatively small, ethnically homogenous, and originated from a single center, with a modest control group. The analyzed FFPE samples (collected between 2009 and 2015) may be subject to potential antigen loss despite appropriate storage condition [39]. In addition, IHC alone provides only semi-quantitative information, and the analysis was restricted to IL-8, NF-kB and Hsp90 expression, without integrating other relevant molecular alterations such as *FGFR3* or *TP53*. These factors limit the generalizability of our findings and highlight the need for validation in larger, more diverse cohorts using complementary functional approaches.

## Conclusions

This study highlights the potential of Hsp90, particularly the Hsp90β isoform, as a biomarker in urothelial carcinoma, with expression correlating with stage, grade, and nodal status. While NF-κB and IL-8 did not show consistent prognostic value in our cohort, their expression underscores the contribution of inflammatory signaling to tumor biology. Incorporating Hsp90β into prognostic models may improve risk stratification and guide therapeutic decision-making. These findings warrant further validation in larger cohorts and support ongoing exploration of Hsp90-targeted strategies in UC.

## Supplementary Information

Below is the link to the electronic supplementary material.

Supplementary file1 (DOCX 115 kb)

## Data Availability

The datasets generated and analyzed during the current study can be obtained from the corresponding author upon reasonable request.
