# Supplementary material for: HSP90β as a potential prognostic biomarker in urothelial carcinoma: insights from NF-κB, IL-8, and heat-shock protein expression
Source: Clin Transl Oncol. 2025 Dec 23;28(6):2335–44. doi: 10.1007/s12094-025-04181-9 (PMC13186835; doi:10.1007/s12094-025-04181-9)

**Supplementary Tabler 1.** Basic characteristics of the TCGA cohort

| Clinical data | | TCGA GDC BLCA, n(%) |
| --- | --- | --- |
| Cases |  | 403 |
| Median age |  | 68.5 (range 34-89) |
| Sex | Female | 106 (26.3%) |
|  | Male | 297 (73.7%) |
| Race | White | 330 (79.4%) |
|  | Black or African-American | 23 (5.7%) |
|  | Asian | 43 (10.7%) |
| Clinical stage | I | 9 (2.3%) |
|  | II | 111 (27.3%) |
|  | III | 30 (7.4%) |
|  | IV | 11 (2.7%) |
| Tumor stage | T2 | 118 (29.3%) |
|  | T3 | 194 (48.1%) |
|  | T4 | 57 (14.1%) |
| Lymph nodes invasion | N0 | 235 (58.3%) |
|  | N1-N3 | 11 (2.7%) |
| Metastasis | M0 | 192 (47.6%) |
|  | M1 | 128 (31.8%) |
| Follow-up time (median) |  | 17.3 months (range 0-166) |
| Survival status | Alive | 224 (55.6%) |
|  | Dead | 179 (44.4%) |

Due to missing data, no cases not always sum up to 403.

**Supplementary Table 2.** The correlation of clinicopathological features of bladder cancer and HSP90AB1 in the TCGA cohort

| **Variables** | **Total (N, %)** | **Median HSP90AB1 expression (FPKM)** | **Q1** | **Q3** | **P value** |
| --- | --- | --- | --- | --- | --- |
| **Gender** |  |  |  |  |  |
| **Females** | 106 (26.3%) | 9.1 (range 7-10.3) | 8.7 | 9.4 | 0.76 |
| **Males** | 297 (73.7%) | 9.1 (range 5.9-11.2) | 8.7 | 9.4 |  |
| **Race** |  |  |  |  |  |
| **White** | 330 (79.4%) | 9.1 (range 7.6-11.1) | 8.7 | 9.4 |  |
| **Black or African American** | 23 (5.7%) | 9.2 (range 7.8-10.3) | 8.6 | 9.4 |  |
| **Asian** | 43 (10.7%) | 9.1 (range 5.9-11.2) | 8.7 | 9.3 |  |
| **pT status** |  |  |  |  |  |
| **T2** | 118 (29.3%) | 9.2 (range 7-11.2) | 8.8 | 9.4 | 0.92 |
| **T3** | 194 (48.1%) | 9.1 (range 5.9-10.7) | 8.7 | 9.4 |  |
| **T4** | 57 (14.1%) | 9 (range 7.6-11.1) | 8.7 | 9.3 |  |
| **pN status** |  |  |  |  |  |
| **N0** | 235 (58.3%) | 9.1 (range 5.9-10.7) | 8.7 | 9.4 | 0.74 |
| **N1** | 46 (11.4%) | 9.1 (range 8.5-10.7) | 8.9 | 9.4 |  |
| **N2** | 75 (18.6%) | 9.0 (range 7.6-11.1) | 8.7 | 9.4 |  |
| **N3** | 7 (1.7%) | 8.7 (range 8.3-10) | 8.5 | 9.8 |  |
| **Stage** |  |  |  |  |  |
| **I** | 9 (2.3%) | 9.1 (range 5.9-11-2) | 8.5 | 9.3 | 0.34 |
| **II** | 111 (27.3%) | 9.2 (range 7.7-10.7) | 8.8 | 9.5 |  |
| **III** | 30 (7.4%) | 9.1 (range 7.8-10.3) | 8.8 | 9.3 |  |
| **IV** | 11 (2.7%) | 8.7 (range 8.1-10.2) | 8.3 | 9.3 |  |

**Supplementary Figure 1.** HSP90AB1 expression in tumor and normal samples (TCGA)**
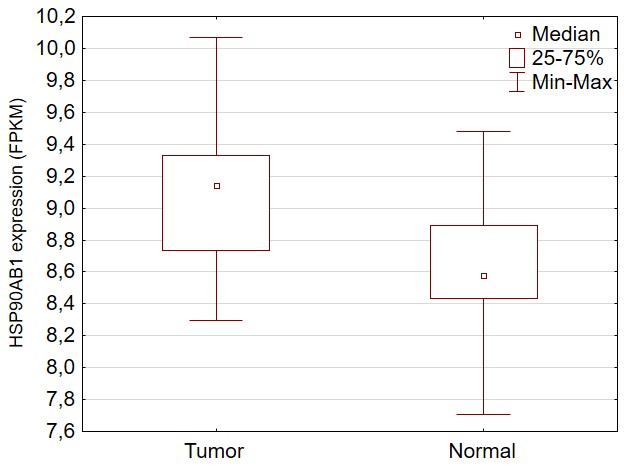
**

**Supplementary Figure 2.** Overall survival in the TCGA laryngeal cancer cohort based on HSP90AB1 expression


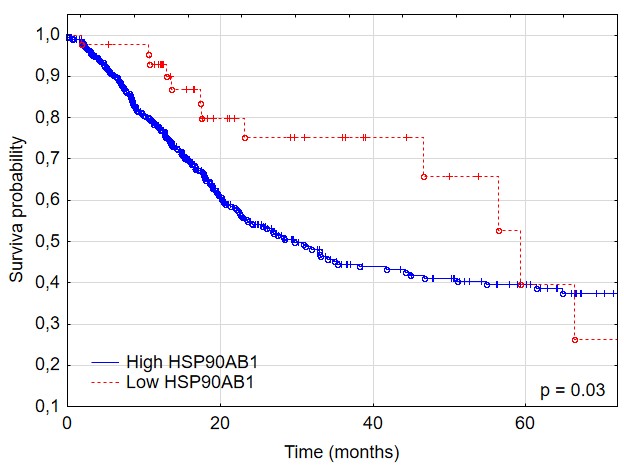

Supplement: Supplementary file 1 — Supplementary file1 (DOCX 115 kb) [file 12094_2025_4181_MOESM1_ESM.docx]
